# Relationship Between Understory Plant Diversity and Soil Physicochemical Properties in Four Vegetation Restoration Forest Types in the Latosol Gully Erosion Area on Hainan Island

**DOI:** 10.3390/plants15182760

**Published:** 2026-09-09

**Authors:** Yanping Huang, Yihan Zhao, Ruowen Mao, Liangying Wu, Yuxian Shen, Yijun An, Jinhui Chen, Zhihua Tu

**Affiliations:** 1 School of Ecology, Hainan University, Haikou 570228, China; 25110713000049@hainanu.edu.cn; 2 Key Laboratory of Genetics and Germplasm Innovation of Tropical Special Forest Trees and Ornamental Plants, Ministry of Education, School of Tropical Agriculture and Forestry, Hainan University, Haikou 570228, China; 24210907000005@hainanu.edu.cn (Y.Z.); 26210907000009@hainanu.edu.cn (R.M.); 24220954000052@hainanu.edu.cn (L.W.); 20233004240@hainanu.edu.cn (Y.S.); 20233004267@hainanu.edu.cn (Y.A.); jinhuichen@hainanu.edu.cn (J.C.); 3 Engineering Research Center of Rare and Precious Tree Species in Hainan Province, Haikou 570228, China

**Keywords:** latosol region, gully erosion, vegetation restoration, understory plant diversity, soil physicochemical properties

## Abstract

Studying the relationships between understory plant diversity and soil physicochemical properties aids in understanding the sustainable development of plantation forests. Vegetation restoration in latosol gully erosion areas plays a key role in preventing soil erosion. Variations in understory plant diversity and soil physicochemical properties in areas that have undergone vegetation restoration subsequent to gully erosion are not well understood. In this study, we investigated the understory species composition, importance values, plant diversity, and soil physicochemical properties and explored their correlations following vegetation restoration using four forest types (*Acacia mangium* forest, *Eucalyptus robusta* forest, *A. mangium–E. robusta* mixed forest, and *A. mangium–E. robusta–Schizostachyum pseudolima* mixed forest) in the Mahuangling Watershed, Hainan Province. A total of 49 plant species belonging to 47 genera and 20 families were recorded. The *E. robusta* forest (31 species) and *A. mangium–E. robusta* mixed forest (27 species) had higher species richness and more complex community structures. The dominant shrub species were *Rhodomyrtus tomentosa*, *Breynia fruticosa*, *Aporosa dioica*, and *Dodonaea viscosa*, while the dominant herbaceous species were *Ageratum conyzoides*, *Chromolaena odorata*, *Spermacoce alata*, and *Erigeron sumatrensis*. In all four vegetation restoration forest types, the richness index in the herbaceous layer was higher than in the shrub layer, while the diversity index showed no significant difference between the shrub and herbaceous layers (*p* > 0.05). The soil bulk density ranged from 1.57 g·cm^−3^ to 1.63 g·cm^−3^, with the *A. mangium* forest having better soil total porosity (38.77%) and water-holding capacity (190.37 t·hm^−2^) than the other forests. NH_4_^+^-N and NO_3_^−^-N were lower in the *E. robusta* forest, while the *A. mangium* forest had significantly higher organic matter content (13.71 g·kg^−1^). Correlation and redundancy analyses showed that soil water content, pH, and soil organic matter were key factors affecting herbaceous-layer and shrub-layer plant diversity. On the whole, we suggest that the planting of pure and mixed forests of *A. mangium* should be considered in future ecological restoration projects in the gully erosion area of Mahuangling in order to maintain the stability of understory plant diversity and improve latosol soil fertility.

## 1. Introduction

Gully erosion is a form of soil erosion generally resulting from a combination of water and gravity effects. It can lead to soil fertility degradation, water eutrophication, and vegetation degradation, posing severe threats to human life and property [1,2,3]. Vegetation restoration, as an effective measure to mitigate soil erosion, is a water and soil conservation measure used in soil erosion areas [2]. Restoring vegetation by planting or enclosure to enhance surface coverage and improve the ecological environment can reduce soil erosion and restore ecosystem functions [2,4]. Due to significant differences in species composition and vegetation structure, the ecological effects of vegetation restoration in different soil erosion areas can vary [5,6].

Vegetation restoration in soil erosion areas plays a key role in improving soil fertility, conserving water and soil, reducing flow and sediment, promoting soil nutrient cycling, and maintaining the stability of plant diversity [7,8,9,10]. Plant diversity is affected by topography, tree species composition, human interference, climate, and other factors [11,12,13,14]. An increasing number of studies show that soil factors are the main elements affecting species and phylogenetic diversity [9,11,14,15]. The planting of indigenous tree species can increase plant diversity and play an important role in maintaining ecosystem multifunctionality [16]. Hydrothermal factors influence the spatial distribution patterns of species diversity [17]. During vegetation restoration, plant species richness, importance value, and plant community diversity show a trend of increasing as the restoration area expands [18]. Vegetation restoration can significantly improve soil quality, which in turn affects understory plant diversity, and their synergistic changes have a positive effect on ecosystem stability [19,20,21]. Different stand types, stand densities, stand ages, restoration degrees, and forest management measures significantly affect plant diversity and soil physicochemical properties [18,22,23,24,25]. Some studies have shown that soil organic matter, total nitrogen, total phosphorus, and available potassium are the main factors affecting the Shannon–Wiener diversity index of understory plants [26]. As stand density increases, the diversity index of herbaceous layer plant communities increases; it is significantly positively correlated with soil total nitrogen, total phosphorus, alkaline-hydrolyzable nitrogen, available phosphorus, and available potassium [27]. Soil physicochemical properties have primarily indirect effects on plant diversity. Introducing appropriate tree species in soil erosion areas can further improve the natural regeneration of forests, thereby supporting sustainable forest management [28], promoting the growth of understory vegetation and the accumulation of soil nutrients [29,30], and driving improvements in soil functionality [31].

The eroded latosol areas in the Mahuangling Watershed in Danzhou County, Hainan Province, are a consequence of the extensive destruction of natural forests from the 1960s to the 1980s, which resulted in severe soil and water erosion during the rainy season. The Mahuangling Watershed is now known as the “Hainan Loess Plateau” and contains numerous erosion gullies, the deepest of which reaches 30 m, causing serious ecological and environmental damage [2,32,33]. Since 2000, the Hainan government has implemented large-scale vegetation restoration projects, including planting *Acacia mangium* and *Eucalyptus robusta* trees, among others. With these efforts, water and soil loss have been effectively controlled, which has gradually improved the ecological environment in the Mahuangling Watershed [2,33]. Barren soil in the gully erosion region that underwent restoration, however, causes frequent collapses of the gully banks, and a risk of further erosion and expansion remains, which has been more challenging to address than expected.

Vegetative restoration was performed in the Mahuangling Watershed’s gully erosion areas using four types of forest: *Eucalyptus robusta* forest, *Acacia mangium* forest, *A. mangium–E. robusta* mixed forest, and *A. mangium–E. robusta–Schizostachyum pseudolima* mixed forest. As a result, gully erosion was effectively controlled, and the regional environment was improved. We previously studied the water conservation characteristics of vegetation restoration in these gully erosion areas. However, how vegetation restoration improves understory plant diversity and soil physicochemical properties remains poorly understood. In this study, we investigated the effects of restoration using four forest types in the latosol gully erosion areas of the Mahuangling Watershed. We explored the understory species composition and plant diversity in the shrub and herbaceous layers and quantified the soil physical and chemical properties under different vegetation restoration approaches, revealing their relationships. We aimed to answer the following research questions: (1) How do different vegetation restoration forest types affect understory plant species composition and diversity? (2) What are the differences in soil physicochemical properties among these restoration types? (3) Which soil physicochemical factors are the primary drivers of understory plant diversity in this specific latosol gully erosion area? The results are expected to be useful in illuminating the effects of vegetation restoration on ecological quality, thus providing a theoretical and practical basis for vegetation restoration and sustainable management in these latosol gully erosion areas.

## 2. Materials and Methods

### 2.1. Study Sites

**Figure 1:**
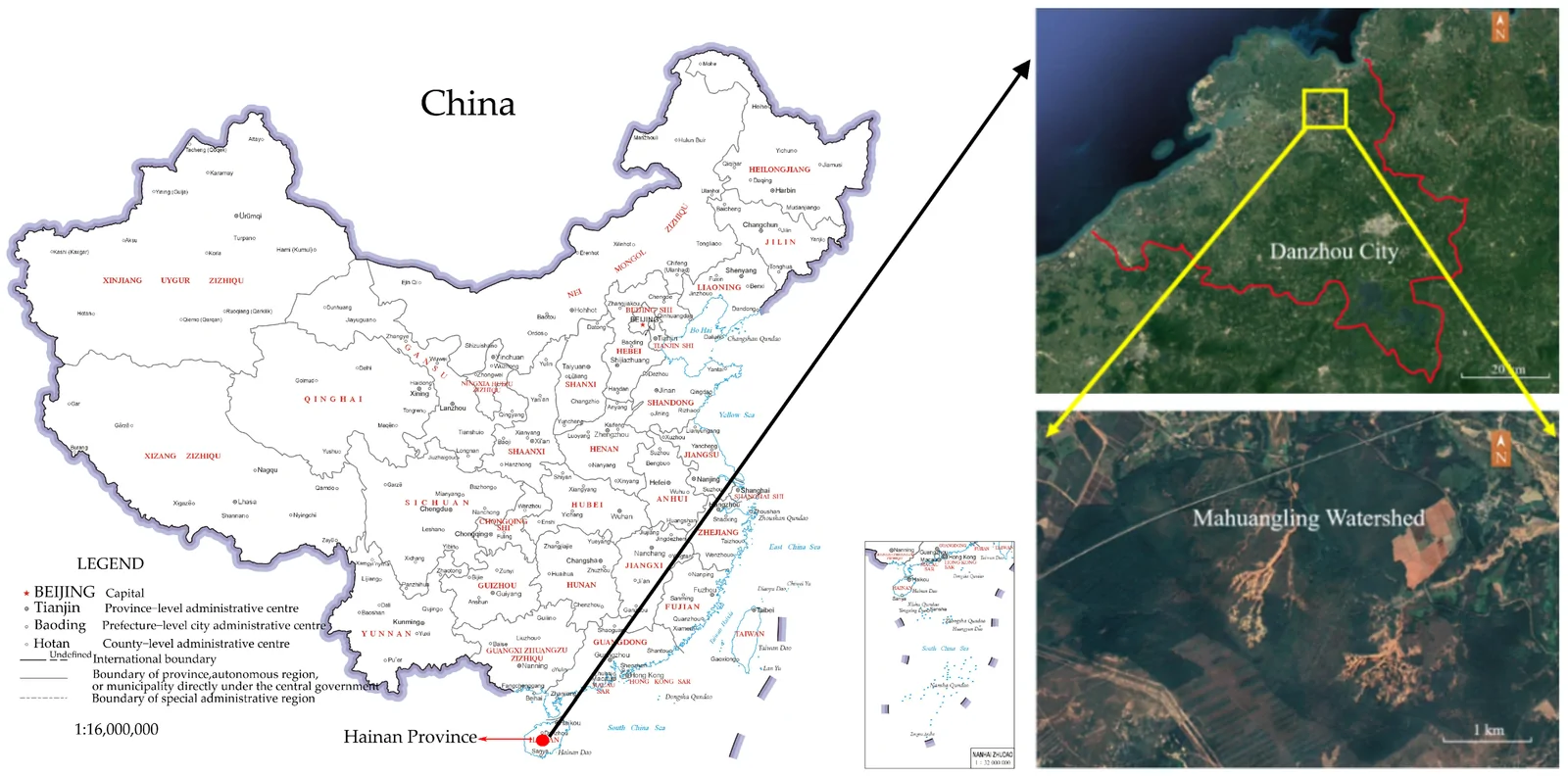
The locations of the sampling sites at the Mahuangling Soil and Water Conservation Monitoring Station.

This study was conducted in the Mahuangling Watershed at the Mahuangling Soil and Water Conservation Monitoring Station (19°41′~19°47′ N, 109°24′~109°30′ E) (Figure 1), located in Danzhou City, Hainan Province, China. The region has a tropical monsoon climate, with a mean annual temperature of 23.5 °C. The mean annual rainfall is approximately 1815 mm, with about 80%~85% occurring from May to October. The soils are typical latosol soils that originated from granitic parent materials. Historically, due to vegetation over-harvesting and deforestation from the 1960s to the 1980s, soil erosion intensified in the already eroded latosol area, leading to soil structural destruction and soil fertility decline across the study area; thus, the Mahuangling Watershed is now known as the “Hainan Loess Plateau” with numerous erosion gullies, the deepest of which reaches 30 m, causing serious ecological and environmental damage [2,32,33]. In the 2000s, vegetation restoration was conducted within the gully erosion area as part of a government-led vegetation restoration project [2,33], including the plantation of *E. robusta* forest, *A. mangium* forest, *A. mangium–E. robusta* mixed forest, and *A. mangium–E. robusta–Schizostachyum pseudolima* mixed forest. All vegetation restoration forest types with similar restoration years are located within the same watershed, on the same soil type, and under identical climatic conditions. The consistent degraded baseline, geographical environment, climatic conditions, and restoration background ensure the comparability of the four vegetation restoration forest types. These restoration efforts have proven successful in controlling soil and water loss, greatly improving the local environment. Moreover, this study site is now primarily covered by plantation forests. The understory consists of species such as *R. tomentosa*, *B. fruticosa*, *A. dioica*, *D. viscosa*, *J. elongatum, P. thunbergii*, *P. repens*, *A. conyzoides*, *C. odorata*, and *S. calendulacea*, and the forest coverage is more than 75% [2,33].

### 2.2. Plot Setting and Survey

Field surveying and soil sampling were conducted in July 2024, which is in the rainy season with sufficient hydrothermal conditions. During this period, understory shrubs and herbaceous plants grow rapidly, with complete species composition and stable community structure, objectively and accurately reflecting understory plant diversity in the restoration forest. Meanwhile, the soil water content, nutrient cycling, and soil physicochemical properties remain relatively stable in the vigorous growing season. Three 10 m × 40 m plots were established within each forest type, located in areas away from the road and relatively free from human disturbance. A total of 12 plots were established. In each plot, we measured the tree height, the trunk diameter at breast height (1.3 m), and the canopy density, as well as recording the elevation, slope aspect, and latitude and longitude of each plot. In each plot, five shrub quadrats of 5 m × 5 m were set up, within which all shrub plants were surveyed, with the species name, number of plants, ground diameter, and height of the shrub layer recorded (H < 3 m, including young seedlings of understory trees). Simultaneously, five herbaceous quadrats of 1 m × 1 m were set up in each plot, and all herbaceous plants within the quadrat were surveyed, with their species name, height, and cover of the herb layer recorded. Basic information for each plot is reported in Table 1.

### 2.3. Soil Sample Collection and Determination

The soil samples were collected at soil layer depths of 0 to 10 cm, 10 to 20 cm, 20 to 40 cm, and 40 to 60 cm from soil profiles using the cutting ring (100 cm^3^) method. Three soil profiles were randomly selected for each plot, and soil was extracted from each layer with two cutting rings. A total of 288 cutting rings (4 forest types × 3 plots × 3 soil profiles × 4 soil layers × 2 cutting rings) were collected for the determination of soil physical properties (soil bulk density, soil capillary porosity, total soil porosity, soil water content, soil maximum water-holding capacity, and soil effective water-holding capacity). Meanwhile, 1 kg of soil from each soil layer was collected, sieved through a 2 mm sieve, placed in a sealable bag, and transported to the laboratory for the determination of soil chemical properties. A total of 144 fresh soil samples (4 forest types × 3 plots × 3 soil profiles × 4 soil layers × 1 soil sample) were collected. Fresh soil samples were brought back to the laboratory and divided into 2 parts: one fresh soil sample was used to measure soil NH_4_^+^-N and NO_3_^−^-N, while the other was air-dried and used for the measurement of soil organic matter and pH.

The oven-drying method was used to determine the soil water content. The cutting ring method was used to determine the soil bulk density, capillary porosity, and total porosity [2,34]. The potentiometric method (Soil Testing Part 2: Method for determination of soil pH, NY/T 1121.2-2006 [35]) was used to determine the soil pH (in a water-to-soil ratio of 2.5:1). The potassium dichromate–sulfuric acid digestion method (Soil Testing Part 6: Method for determination of soil organic matter, NY/T 1121.6-2006 [36]) was used to determine the soil organic matter content. Extraction with potassium chloride solution and a manual method (Soil quality—Determination of nitrate, nitrite and ammonium in soils—Extraction with potassium chloride solution and determination with manual method, GB/T 42485-2023 [37]) were used to determine the contents of NH_4_^+^-N and NO_3_^−^-N.

### 2.4. Determination of Plant Community Species Diversity Index

Community species diversity was measured [38] as presented below.

The richness index (*S*):

*S* = N (1)

The Shannon–*Wiener* index (*H*):

(2) $$H\;=\;\sum_{i=0}^{s}P_{i}lnP_{i}$$

The Simpson index (*D*):

(3) $$D\;=\;\sum_{i=0}^{s}{\left(P_{i}\right)}^{2}$$

The Pielou index (*Jh*):

(4) Jh = HlnS

In the formula, *P_i_* = *n_i_*/N, N is the total number of species, *n_i_* is the *i*th species, and *ln* is the natural logarithm based on *e*.

### 2.5. Statistical Analysis

Differences in the understory plant diversity index and soil physicochemical properties of the four vegetation restoration forest types were analyzed using a one-way analysis of variance (ANOVA). The least significant differences (LSDs) were determined for multiple comparisons. The significance level was set at *p* < 0.05. The above analyses were performed using SPSS v. 27.0 (SPSS Inc., Chicago, IL, USA). The Origin 2021 software (Origin, Origin Lab, Farmington, ME, USA) was used to plot the figures, and the correlation plot in Origin was used to perform Pearson correlation analysis on the understory vegetation species diversity index and soil physicochemical properties and to plot the correlation heatmap. Canoco 5.0 was used for the redundancy analysis (RDA) of understory plant diversity with soil physicochemical properties, which directly quantified the proportion of variance in plant diversity that is explained by soil factors.

## 3. Results

### 3.1. Species Composition of Understory Plants

The species composition of the surveyed shrub and herbaceous layers of the understory plants in the Mahuangling Watershed was relatively simple. A total of 49 plant species belonging to 47 genera and 20 families were recorded and investigated. The *E. robusta* forest had 31 plant species belonging to 31 genera and 15 families; the *A. mangium* forest had 16 plant species belonging to 16 genera and 10 families; the *A. mangium–E. robusta* mixed forest had 27 plant species belonging to 26 genera and 14 families; and the *A. mangium–E. robusta–S. pseudolima* mixed forest had 24 plant species belonging to 23 genera and 11 families (Table 2). For the number of families, genera, and species in the understory plants, the four vegetation restoration forest types ranked as follows: *E. robusta* forest > *A. mangium–E. robusta* mixed forest > *A. mangium–E. robusta–S. pseudolima* mixed forest > *A. mangium* forest.

**Figure 2:**
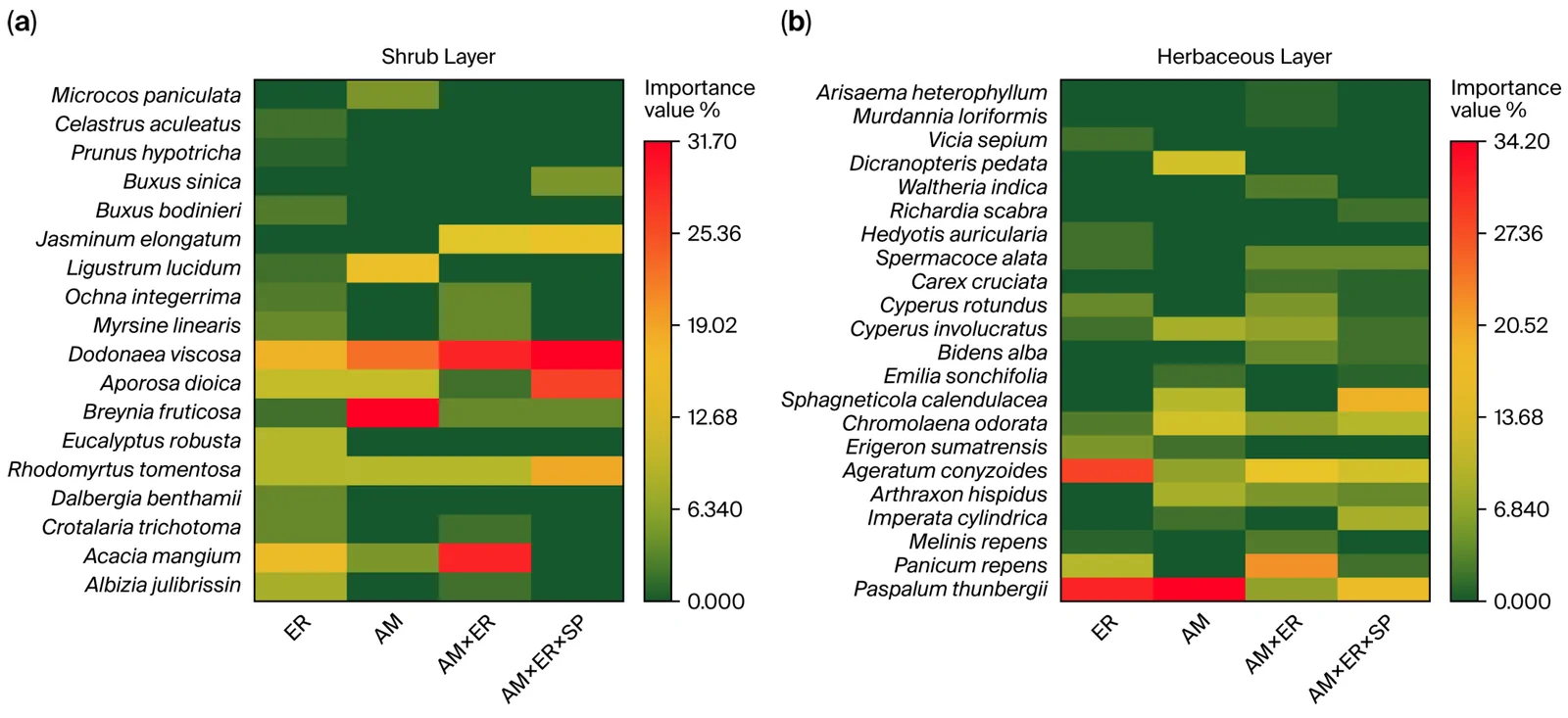
The importance values of shrub- and herbaceous-layer plant species of different vegetation restoration forest types: (**a**) shrub layer; (**b**) herbaceous layer. *E. robusta* forest (ER), *A. mangium* forest (AM), *A. mangium–E. robusta* mixed forest (AM × ER), and *A. mangium–E. robusta–S. pseudolima* mixed forest (AM × ER × SP).

In all four vegetation restoration forest types, the shrub layer consisted of 18 plant species belonging to 17 genera and 11 families, including dominant species such as *Rhodomyrtus tomentosa*, *Breynia fruticosa*, *Aporosa dioica*, *Dodonaea viscosa*, and *Jasminum elongatum*. Among them, the importance values of *R. tomentosa* in the four vegetation types, *E. robusta* forest, *A. mangium* forest, *A. mangium–E. robusta* mixed forest, and *A. mangium–E. robusta–S. pseudolima* mixed forest, were 8.56%, 9.12%, 8.56%, and 18.09%, respectively; those of *B. fruticosa* were 2.47%, 30.76%, 4.74%, and 4.34%, respectively; those of *A. dioica* were 10.21%, 10.15%, 2.90%, and 25.69%, respectively; those of *D. viscosa* were 17.33%, 22.79%, 28.51%, and 31.70%, respectively; and those of *J. elongatum* in the two mixed forests were 11.90% and 14.59%, respectively (Figure 2a).

The herbaceous layer consisted of 31 plant species belonging to 30 genera and 11 families, including *Paspalum thunbergii*, *Panicum repens*, *Ageratum conyzoides*, *Chromolaena odorata*, and *Sphagneticola calendulacea*. Among them, the importance values of *P. thunbergii* in the four vegetation types were 29.99%, 34.11%, 7.45%, and 17.20%, respectively; those of *A. conyzoides* were 28.46%, 7.52%, 14.40%, and 11.95%, respectively; and those of *C. odorata* were 3.43%, 12.39%, 7.89%, and 10.00%, respectively. The importance value of *P. repens* was highest in the *A. mangium–E. robusta* mixed forest, reaching 22.41%, while that of *S. calendulacea* was the highest in the *A. mangium–E. robusta–S. pseudolima* mixed forest, reaching 18.97% (Figure 2b).

Overall, the understory plant community structures of the four vegetation restoration forest types are fairly similar, with fewer plant species and a simpler community structure compared with the total of 49 species recorded. Grass species occupy the dominant ecological niche in the herbaceous layer. Additionally, in each forest type, the herbaceous layer was primarily composed of invasive species such as *A. conyzoides*, *C. odorata*, *S. calendulacea, Spermacoce alata*, and *Erigeron sumatrensis*, suggesting that the harsh gully erosion environment, characterized by fragmented terrain and water stress, favors disturbance-tolerant, fast-spreading species with low environmental requirements while limiting the establishment of more specialized or late-successional species.

### 3.2. Understory Plant Diversity of Different Vegetation Restoration Forest Types

**Figure 3:**
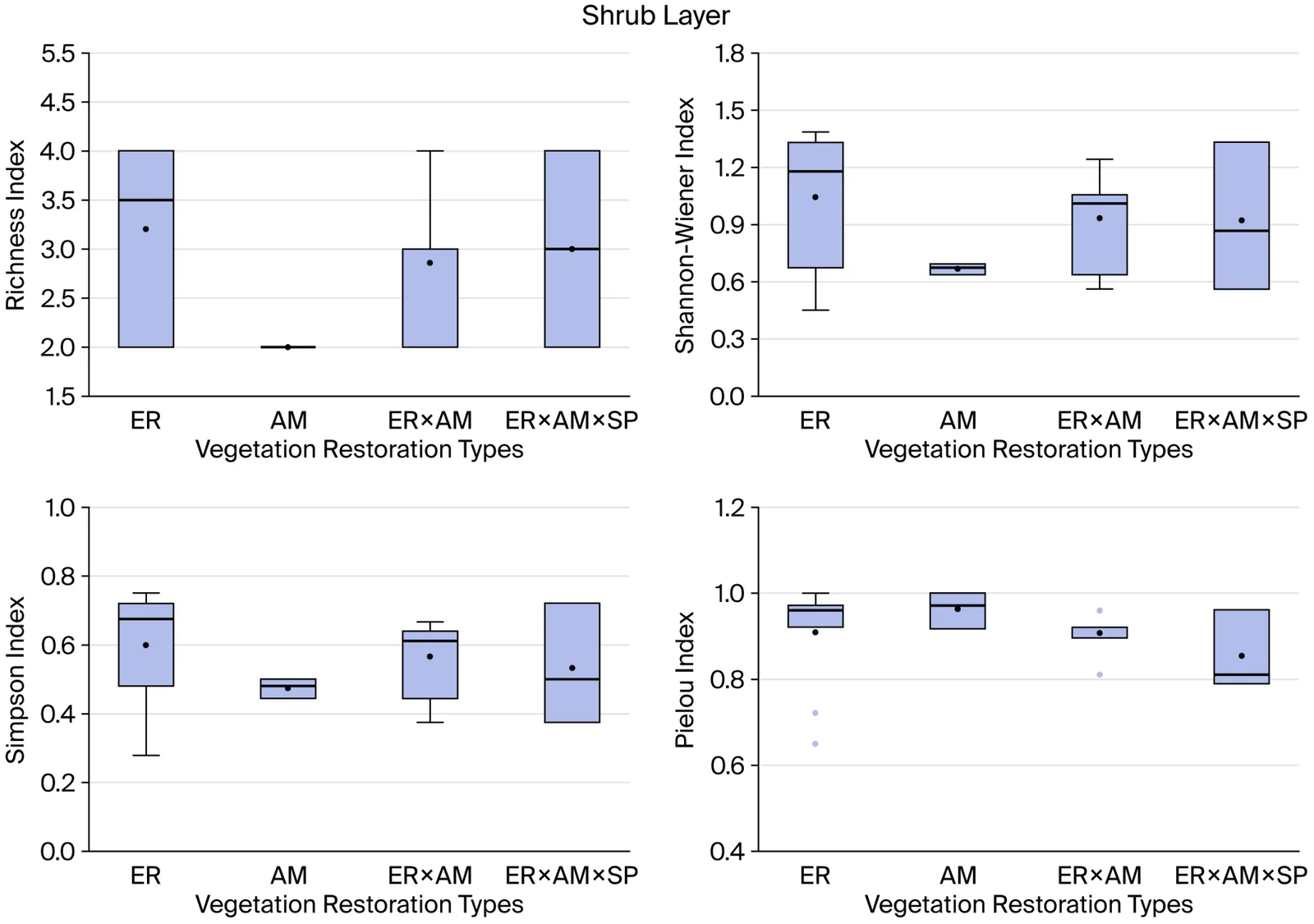
Plant diversity index of shrub layer in different vegetation restoration forest types. *E. robusta* forest (ER), *A. mangium* forest (AM), *A. mangium–E. robusta* mixed forest (AM × ER), and *A. mangium–E. robusta–S. pseudolima* mixed forest (AM × ER × SP).

The species diversity values of the shrub and herbaceous layers in four vegetation restoration forest types are shown in Figure 2 and Figure 3. For the shrub layer, the richness index (3.20), Simpson index (0.60), and Shannon–*Wiener* index (1.04) of *E. robusta* forest were the highest, while those of *A. mangium* forest were the lowest (richness index 2.00, Simpson index 0.47, and Shannon–*Wiener* index 0.67). The Pielou index was highest in *A. mangium* forest (0.96) and lowest in *A. mangium–E. robusta–S. pseudolima* mixed forest (0.85) (Figure 3). This indicates that the shrub layer in *E. robusta* forest has more understory plant species, while *A. mangium* forest has fewer, but the distribution is more evenly spread.

**Figure 4:**
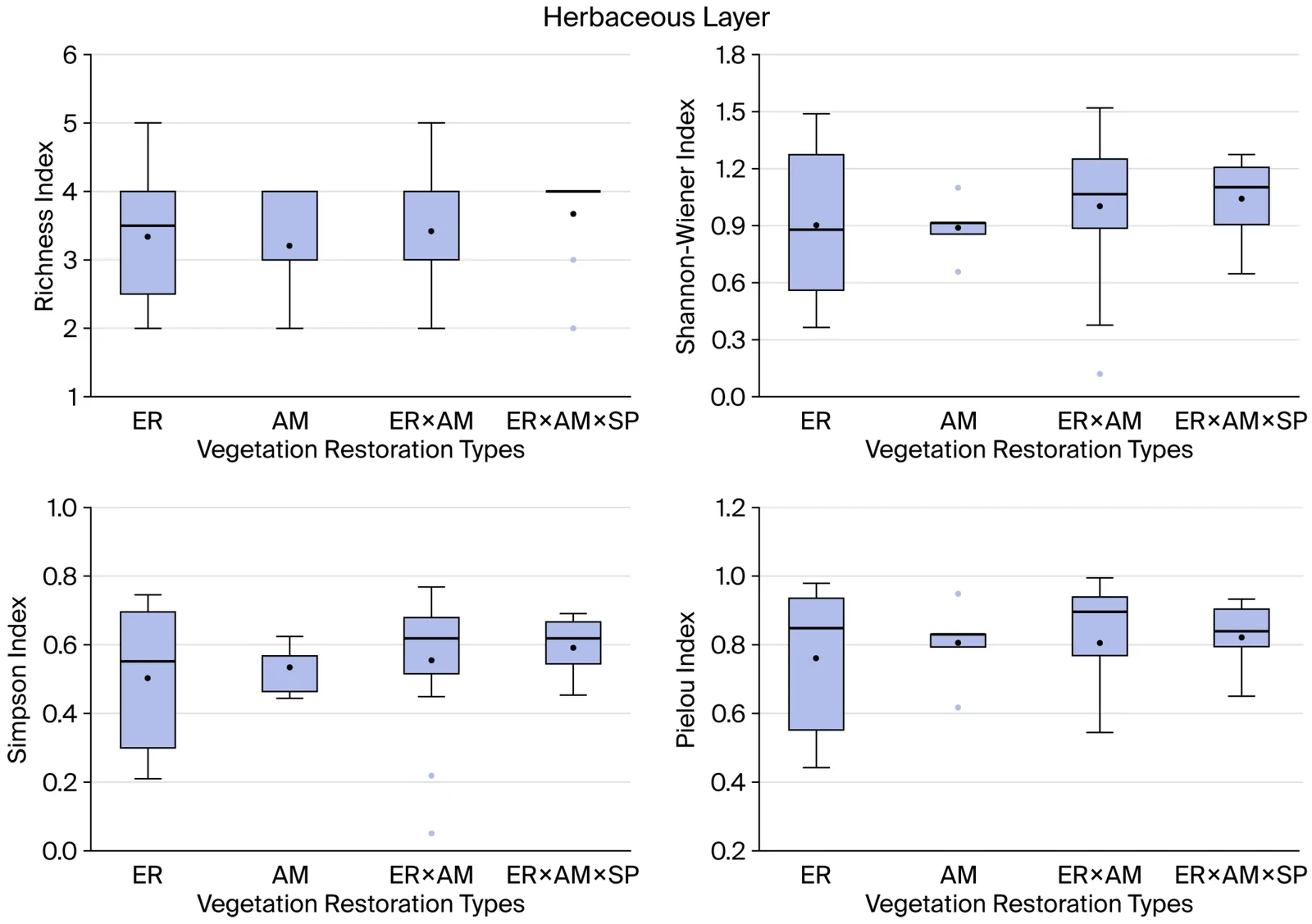
Plant diversity index of the herbaceous layer in different vegetation restoration forest types. *E. robusta* forest (ER), *A. mangium* forest (AM), *A. mangium–E. robusta* mixed forest (AM × ER), and *A. mangium–E. robusta–S. pseudolima* mixed forest (AM × ER × SP).

For the herbaceous layer, the richness index (3.67), Simpson index (0.59), Shannon–*Wiener* index (1.04), and Pielou index (0.82) of *A. mangium–E. robusta–S. pseudolima* mixed forest were the highest, while those of *A. mangium* forest (richness index 3.20 and Shannon–*Wiener* index 0.89) and *E. robusta* forest (Simpson index 0.50 and Pielou index 0.76) were the lowest (Figure 4). This indicates that *A. mangium–E. robusta–S. pseudolima* mixed forest has more understory herbaceous plant species and a more uniform distribution.

Overall, across the four vegetation restoration forest types, the herbaceous layer consistently exhibited higher species richness than the shrub layer, while the two layers showed comparable diversity (Shannon–Wiener) and evenness (Pielou) indices (*p* > 0.05). Among the restoration types, *E. robusta* forest supported the highest shrub diversity, whereas the *A. mangium–E. robusta–S. pseudolima* mixed forest supported the highest herbaceous diversity; meanwhile, *A. mangium* forest had the lowest diversity in both layers but exhibited the highest shrub evenness. These results suggest that the vegetation restoration forest type influences understory diversity primarily through species richness rather than community evenness and that the overall shrub–herbaceous community structure remains relatively stable across different restoration types in the gully erosion environment.

### 3.3. Soil Physicochemical Properties of Different Vegetation Restoration Forest Types

The soil physical properties differed significantly among the four vegetation restoration forest types (*p* < 0.05). In the 0 to 60 cm soil layer, the soil bulk density ranged from 1.57 g·cm^−3^ to 1.63 g·cm^−3^, which was lowest in the *A. mangium* forest, and there was no significant difference observed between *A. mangium–E. robusta* mixed forest and *A. mangium–E. robusta–S. pseudolima* mixed forest. The total soil porosity was 38.77%, 35.38%, 34.14%, and 34.09% for *A. mangium* forest, *A. mangium–E. robusta–S. pseudolima* mixed forest, *E. robusta* forest, and *A. mangium–E. robusta* mixed forest, respectively (Table 3). The *A. mangium* forest had the highest soil capillary porosity (35.91%), followed by *A. mangium–E. robusta–S. pseudolima* mixed forest, *E. robusta* forest, and *A. mangium–E. robusta* mixed forest (31.55%) (Table 3). The soil water content was 8.15%, 6.94%, 4.42%, and 3.59% for *A. mangium–E. robusta–S. pseudolima* mixed forest, *A. mangium–E. robusta* mixed forest, *E. robusta* forest, and *A. mangium* forest, respectively (Table 3). The soil maximum water-holding capacity decreased in the order of *A. mangium* forest (190.37 t·ha^−1^) > *A. mangium–E. robusta–S. pseudolima* mixed forest (177.30 t·ha^−1^) > *E. robusta* forest (174.72 t·ha^−1^) > *A. mangium–E. robusta* mixed forest (172.95 t·ha^−1^). The soil effective water-holding capacity was highest in *A. mangium* forest (176.31 t·ha^−1^), followed by the *E. robusta* forest, *A. mangium–E. robusta–S. pseudolima* mixed forest, and *A. mangium–E. robusta* mixed forest (154.03 t·ha^−1^) (Table 3).

The soil chemical properties differed significantly among different vegetation restoration forest types (*p* < 0.05). The soil pH ranged from 5.06 to 5.09, and the soil organic matter decreased in the order of *A. mangium* forest (13.71 g·kg^−1^) > *A. mangium–E. robusta* mixed forest (9.92 g·kg^−1^) > *A. mangium–E. robusta–S. pseudolima* mixed forest (7.01 g·kg^−1^) > *E. robusta* forest (5.50 g·kg^−1^). The content of soil NH_4_^+^-N was 64.73 mg·kg^−1^, 53.70 mg·kg^−1^, 43.80 mg·kg^−1^, and 35.86 mg·kg^−1^ for *A. mangium–E. robusta–S. pseudolima* mixed forest, *A. mangium–E. robusta* mixed forest, *A. mangium* forest, and *E. robusta* forest, respectively. The content of soil NO_3_^−^-N was highest in the *A. mangium–E. robusta–S. pseudolima* mixed forest (2.78 mg·kg^−1^), followed by the *A. mangium–E. robusta* mixed forest, *A. mangium* forest, and *E. robusta* forest (0.83 mg·kg^−1^) (Table 4).

Overall, significant differences in soil physicochemical properties were found among different vegetation restoration forest types. The total soil porosity, soil capillary porosity, soil maximum water-holding capacity, and soil effective water-holding capacity of the *A. mangium* forest were significantly higher than those of the other vegetation types (*p* < 0.05), indicating that the *A. mangium* forest has better water-holding capacity. The soil water content, soil organic matter, NH_4_^+^-N, and NO_3_^−^-N in the mixed forests were significantly higher than those of the pure forests (*p* < 0.05), indicating that mixed forests are better able to improve soil fertility. Additionally, in the 0 to 60 cm soil layer, the soil bulk density gradually increased with increasing soil depth in all stands, while the total porosity, soil maximum water-holding capacity, soil effective water-holding capacity, soil organic matter, NH_4_^+^-N, and NO_3_^−^-N of the soil gradually decreased with increasing soil depth in all stands.

In summary, the *A. mangium* forest demonstrated superior soil physical properties, while the mixed forests exhibited better soil chemical fertility. These findings highlight a trade-off between physical and chemical soil improvements among different restoration types.

### 3.4. Relationship Between Understory Plant Diversity and Soil Physicochemical Properties

**Figure 5:**
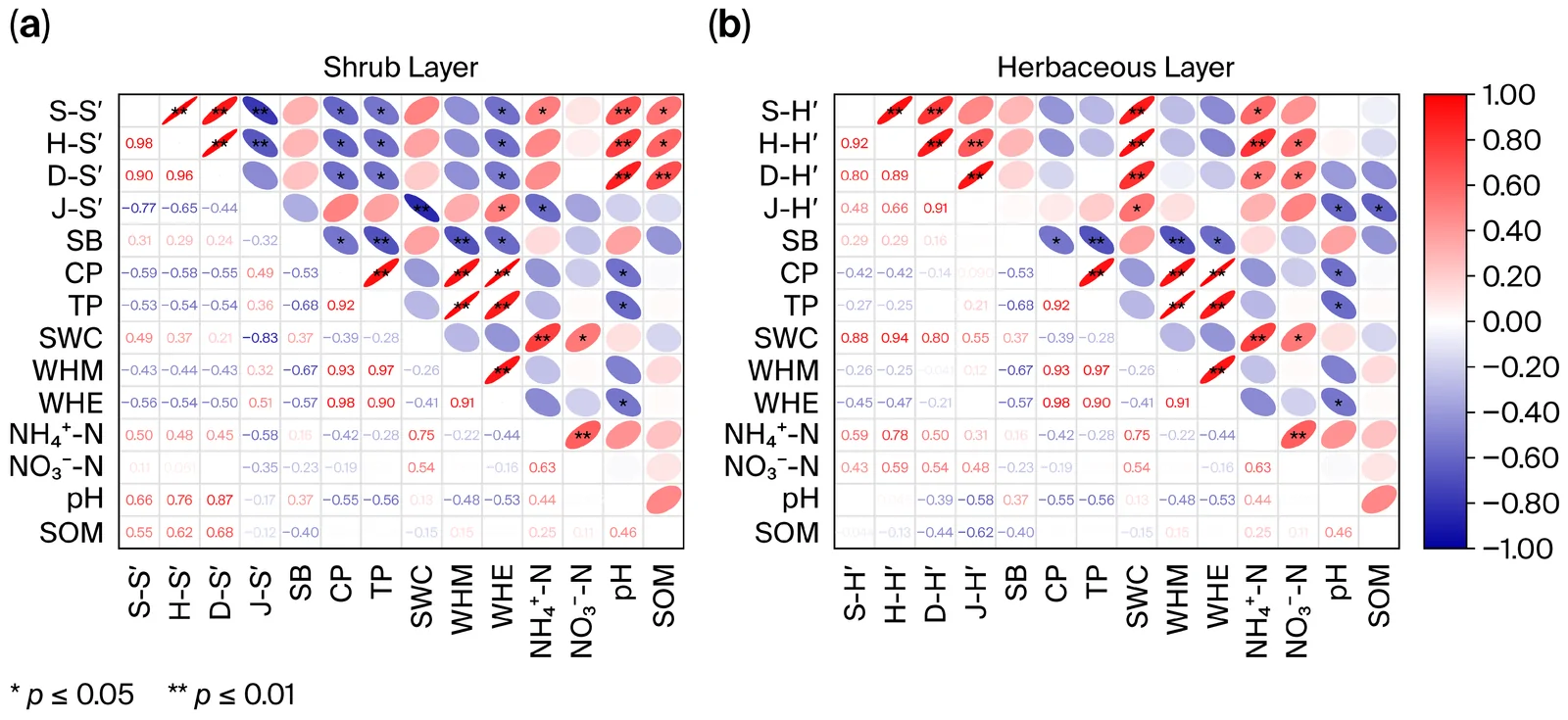
Relationship between understory plant diversity and soil physicochemical properties in four vegetation restoration forest types: (**a**) shrub layer; (**b**) herbaceous layer. S-S’, H-S’, D-S’, J-S’, SB, CP, TP, SWC, WHM, WHE, and SOM, respectively, are the richness index, Shannon–*Wiener* index, Simpson index, Pielou index, soil bulk density, soil capillary porosity, total soil porosity, soil water content, soil maximum water-holding capacity, soil effective water-holding capacity, and soil organic matter.

For the shrub layer, the richness index, Shannon–Wiener index, and Simpson index were significantly negatively correlated with soil capillary porosity, total porosity, and soil effective water-holding capacity (*p* < 0.05) and significantly positively correlated with soil pH value (*p* < 0.01). The richness index and Shannon–Wiener index were significantly positively correlated with soil organic matter content (*p* < 0.05), while the Simpson index was highly significantly positively correlated with soil organic matter content (*p* < 0.01). The Pielou index was significantly negatively correlated with NH_4_^+^-N (*p* < 0.05) and highly significantly negatively correlated with soil water content (*p* < 0.01). The richness index was also significantly positively correlated with NH_4_^+^-N (*p* < 0.05) (Figure 5a).

For the herbaceous layer, the Pielou index was significantly positively correlated with soil water content (*p* < 0.05), and all plant diversity indices were significantly positively correlated with soil water content (*p* < 0.01). The richness index and Simpson index were significantly positively correlated with NH_4_^+^-N (*p* < 0.05), while the Shannon–Wiener index was extremely significantly positively correlated with NH_4_^+^-N (*p* < 0.01). The Shannon–Wiener index and Simpson index were significantly positively correlated with NO_3_^−^-N (*p* < 0.05), while the Pielou index was significantly negatively correlated with soil pH and soil organic matter (*p* < 0.05) (Figure 5b).

**Figure 6:**
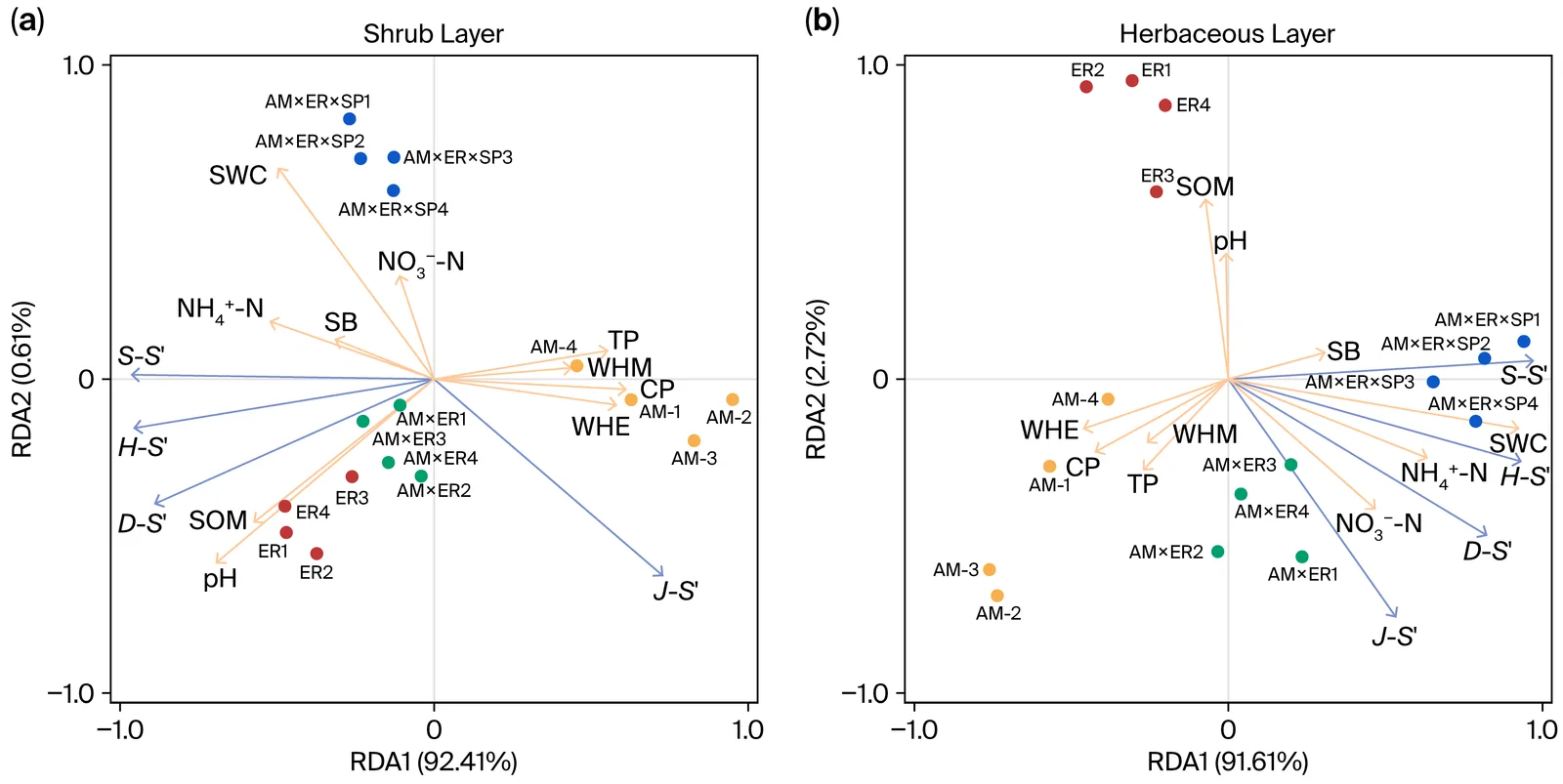
Redundancy analysis (RDA) of understory plant diversity with soil physicochemical properties in four vegetation restoration forest types: (**a**) shrub layer and (**b**) herbaceous layer. S-S’, H-S’, D-S’, J-S’, SB, CP, TP, SWC, WHM, WHE, and SOM, respectively, are the richness index, Shannon–*Wiener* index, Simpson index, Pielou index, soil bulk density, soil capillary porosity, total soil porosity, soil water content, soil maximum water-holding capacity, soil effective water-holding capacity, and soil organic matter. *E. robusta* forest (ER), *A. mangium* forest (AM), *A. mangium–E. robusta* mixed forest (AM × ER), and *A. mangium–E. robusta–S. pseudolima* mixed forest (AM × ER × SP).

To further investigate the effects of soil physicochemical properties on understory plant diversity, we performed a redundancy analysis (RDA) with understory plant diversity as the response variable and the soil physicochemical properties as the explanatory variables (Figure 6a). For the shrub layer, the soil physicochemical factors accounted for 93.02% of the total variance in the shrub layer understory plant diversity. The first and second ranked axes (RDA1 and RDA2) explained 92.41% and 0.61%, respectively, for a cumulative 93.02% of the variance in shrub-layer species diversity (Figure 6a). Among the soil physicochemical factors, pH exhibited the highest explanatory power for shrub-layer species diversity, accounting for 44.60% (*p* = 0.006) of the variance, followed by soil water content (15.90%) (*p* = 0.034), soil organic matter (14.60%) (*p* = 0.016), and NH_4_^+^-N (9.10%) (*p* = 0.022). These four physicochemical factors primarily influenced the understory plant diversity of the shrub layer in the four vegetation restoration forest types.

For the herbaceous layer, the soil physicochemical factors accounted for 94.33% of the total variance in the herbaceous layer understory plant diversity. RDA1 and RDA2 explained 91.61% and 2.72%, respectively, for a cumulative 94.33% of the variance in herbaceous-layer species diversity (Figure 6b). Among the soil physicochemical factors, soil water content was the dominant explanatory factor, which exhibited the highest explanatory power for herbaceous-layer species diversity, accounting for 78.30% (*p* = 0.002) of the variance, followed by soil organic matter (4.90%) (*p* = 0.046). These two physicochemical factors primarily influenced the understory plant diversity of the herbaceous layer in the four vegetation restoration forest types.

The results consistently indicated soil water content, pH, and soil organic matter as the primary factors governing understory plant diversity in both the shrub and herbaceous layers, suggesting that these are the main ecological factors restricting community assembly in the gully erosion environment.

## 4. Discussion

### 4.1. Understory Species Composition of Different Vegetation Restoration Forest Types

In this study, we found that a total of 49 plant species belonging to 47 genera and 20 families comprised the understory species composition across four vegetation restoration forest types in the latosol gully erosion area on Hainan Island. The species composition of understory plants is a comprehensive reflection of the long-term interaction between plants and the environment and forms the basis for formulating protection strategies, restoring degraded ecosystems, and rationally utilizing natural resources [39,40,41,42]. Through the investigation and analysis of four vegetation restoration approaches to ameliorating gully erosion in the Mahuangling Watershed, we found 49 species of understory plants in the four restoration forest types, which is slightly more than the 36 plant species found in gully erosion areas in the dry–hot valley of Yuanmou, Yunnan [39]. This may be related to the hydrothermal conditions in gully erosion regions, which are not suitable for the growth of plants. The role of herbaceous-layer plant species in gully erosion is greater than that of shrub-layer species, which may be due to the combined effects of gully terrain fragmentation and canopy openness. The gully micro-environment favors shallow-rooted, fast-colonizing herbaceous species over deeper-rooted shrubs, which require more stable soil conditions for establishment. Additionally, the relatively low canopy density (0.19–0.28) of the restoration forest types, except for *A. mangium* forest (0.78), allows ample light penetration to the forest floor, further promoting heliophytic herbaceous growth. In contrast, Mao et al. [40] found that the number of shrub-layer plant species was greater than that of herbaceous-layer species in Guangxi Gaofeng Forest Farm and that the number of understory plants in a mixed forest was greater than in a pure forest. This may be related to the sparse vegetation canopy found in gully erosion areas and better light conditions in stands, which promote the growth of heliophytic herbaceous plants. The importance value index is often used to measure the dominance of species in the plant community and can reflect the relative importance of these species and the relationship between plants and the environment [41]. In gully erosion, the dominant species in the understory included *R. tomentosa, B. fruticosa, A. dioica, P. thunbergii, C. odorata,* and *S. calendulacea*, which is consistent with the research of Du et al. [42], indicating that the common dominant species in plant communities on Hainan Island are relatively similar. This suggests that the harsh gully environment, characterized by high solar radiation, seasonal water stress, and nutrient-poor latosol, acts as an ecological filter that selects for disturbance-tolerant, ruderal species with broad environmental tolerances while excluding more specialized or late-successional shrubs [40,43].

### 4.2. Understory Species Diversity of Four Vegetation Restoration Forest Types in Gully Erosion Areas

Plant diversity can reflect the community structure and overall health and stability of forests, and diversity indices are closely related to vegetation’s ecological effects, playing an important role in ecosystem balance and sustainable development [10,44]. In this study, the maximum values of the understory species richness index, Shannon–Wiener index, and Simpson index in the gully erosion forests were 3.67, 1.04, and 0.59, respectively. These values are lower than those reported by Wang et al. [45], who found a richness index of 13.03 and a Shannon–Wiener index of 1.86 in gully erosion areas in North China. The consistently lower diversity indices observed in our gully erosion forests, compared to those reported from North China gullies [45], can be attributed to the combined stress of tropical latosol’s inherent nutrient deficiency, intense seasonal rainfall leading to rapid nutrient leaching, and the fragmented micro-topography that creates frequent soil moisture fluctuations—factors that collectively impose strong environmental filtering on species recruitment and survival. Meanwhile, the understory plant diversity in the *E. robusta* forest was higher than in the mixed forests, which is inconsistent with the findings of Jiang et al. [46] that converting *E. robusta* forest to mixed forests increased understory plant diversity. This is mainly related to the specific water and heat conditions of gullies. Zeng et al. [47] found a greater number of understory plant species in the *A. mangium* forest compared to the *E. robusta* forest, while the results of this study showed that the *E. robusta* forest had more understory species than the *A. mangium* forest. This may reflect a context-dependent outcome—in the highly disturbed gully environment, the relatively open canopy and simple stand structure of *E. robusta* likely reduce competition for light and space, facilitating the establishment of a broader range of heliophytic species. In contrast, the structurally complex mixed forests, while generally beneficial in stable habitats, may intensify interspecific competition for already limited soil water and nutrients under gully conditions, thereby excluding less competitive species. At the same time, the combined effect of fragmented terrain and rainfall erosion in tropical latosol gully erosion leads to poor vegetation growth, which restricts the effectiveness of soil and water conservation measures [48]. Among the four vegetation restoration forest types, the canopy density of the *A. mangium* forest (0.78) was much higher than that of other forests (0.19–0.28), while its plant diversity index was lower. This indicates that understory plant diversity is regulated by canopy density, which not only limits light availability but also reduces the diversity of microhabitats by suppressing understory vegetation through shading and thick litter accumulation that may hinder seed germination. Appropriate canopy density and mixed forests are key conditions for maintaining shrub–herbaceous diversity [43,49].

### 4.3. Soil Physicochemical Properties as Affected by Vegetation Restoration Forest Types

In this study, soil physicochemical properties varied significantly among different vegetation restoration forest types. Overall, the *A. mangium* forest performed better in improving soil physical structure, with significantly higher capillary porosity, total porosity, soil maximum water-holding capacity, and soil effective water-holding capacity compared to the other vegetation restoration forest types. The superior water-holding capacity of the *A. mangium* forest can be attributed to its dense canopy (0.78), which reduces throughfall kinetic energy and surface runoff, promotes litter accumulation, and enhances soil aggregate formation. In contrast, the lower pH observed in the *A. mangium* forest (5.06) is likely due to the acidic nature of litter decomposition, which releases organic acids into the soil. Additionally, the stand density of the *A. mangium* forest was high, which differs from the conclusion of Liu et al. [50] that low-density stands are beneficial for improving soil structure. Furthermore, this study’s results indicated that mixed forests had significantly higher NH_4_^+^-N and NO_3_^−^-N than pure forests, which is consistent with the conclusion of some studies that mixed forests are more effective than pure forests in improving soil physicochemical properties [51,52]. We found that the *A. mangium* forest had a lower soil NH_4_^+^-N content, which may be due to the high volume of litter and slow decomposition rate, resulting in slower nitrogen mineralization and insufficient accumulation of NH_4_^+^-N [53]. It may also be related to the unique habitats and water and heat conditions of gullies, which typically have fragmented terrain and high risks of nitrogen leaching due to soil erosion and rapid water loss [54,55,56]. In our study, the *A. mangium* forest had a significantly higher soil organic matter content than the other forest types, a finding that aligns with those of Zeng et al. [47] from 2024 and He et al. [53] from 2017. This may be due to the abundance of slowly decomposing litter in the *A. mangium* forest, leading to greater soil organic matter accumulation in the surface layer.

### 4.4. Driving Factors of Understory Plant Diversity: Shrub vs. Herbaceous Layers

Understory plant diversity and soil physicochemical properties are closely related, but there are significant differences between the driving factors of the shrub and herbaceous layers. Correlation analysis shows that shrub-layer species diversity (richness index, Shannon–Wiener index, and Simpson index) is significantly negatively correlated with soil porosity and water-holding capacity and significantly positively correlated with soil pH value and soil organic matter content. This is consistent with the research results of Yan et al. [57], who found that the Shannon–Wiener and Simpson indices are highly significantly positively correlated with soil organic matter but highly significantly negatively correlated with soil pH, indicating that soil organic matter accumulation and suitable pH conditions are crucial to maintaining species diversity. The contrast in driving factors between shrub and herbaceous layers observed in our study reflects fundamental differences in their resource acquisition strategies and ecological niches. Shrubs, with their deeper root systems and longer life cycles, are more dependent on stable soil chemical properties, particularly pH and soil organic matter, which regulate long-term nutrient availability and root–soil interactions [57,58]. In contrast, herbaceous plants, with their shallow, fibrous root systems and rapid turnover rates, are more directly responsive to episodic resource pulses, which are readily accessible in the upper soil layers [59]. In gully erosion environments, poor soil structure and low water-holding capacity may allow only highly tolerant shrub species to survive, resulting in lower diversity, as shrub species can survive in micro-environments rich in organic matter and with more suitable pH values [58]. Therefore, soil organic matter content and pH value are key factors limiting shrub-layer diversity. Herbaceous-layer species diversity is highly positively correlated with soil water content and NH_4_^+^-N content. This is similar to the results of Chen et al. [59], who found that soil water content is the main factor affecting plant species. NH_4_^+^-N is a nitrogen source that can be directly absorbed by plants, increasing their NH_4_^+^-N content and directly promoting the growth and establishment of herbaceous plants and thereby increasing plant diversity. The surface soil water content in gully erosion areas changes dramatically, and the roots of herbaceous plants are shallow, increasing their sensitivity to changes in surface soil water and available nutrients. This study revealed that the Pielou index is negatively correlated with NH_4_^+^-N and soil water content in the shrub layer but positively correlated in the herbaceous layer, indicating that shrubs and herbaceous plants have different resource utilization strategies. In the shrub layer, dominant species may be more dominant when resources are scarce, leading to a decrease in evenness, while in the herbaceous layer, the abundance of resources promotes balance among species. Additionally, according to the RDA results, among soil physicochemical factors, soil water content, pH, and soil organic matter had higher explanatory power for the variance of plant diversity information in the shrub and herbaceous layers. The results indicated that in the four forest types used in vegetation restoration in gully erosion areas, the species diversity of the shrub and herbaceous layers was influenced by the soil water content, pH, and soil organic matter, and these may be the primary factors restricting the successful development of shrub–herbaceous plant communities.

## 5. Conclusions

In the latosol gully erosion area of the Mahuangling Watershed of Hainan Province, China, we studied the relationships between understory plant diversity and soil physicochemical properties in four vegetation restoration forest types. We recorded a total of 49 plant species belonging to 47 genera and 20 families. The *E. robusta* forest and the *A. mangium–E. robusta* mixed forest had higher species richness and more complex community structures. The richness index in the herbaceous layer was higher than in the shrub layer, while the diversity index showed no significant difference between the shrub and herbaceous layers. Lower soil bulk density was associated with greater porosity in the *A. mangium* forest, which increases the soil water-holding capacity, thus reducing surface water loss. The *A. mangium* forest had a significantly higher soil organic matter content, while the contents of NH_4_^+^-N and NO_3_^−^-N were higher in the mixed forests, which improved the soil chemical properties in *A. mangium* pure and mixed forests. Understory plant diversity is primarily affected by the interactions among soil physicochemical properties, such as soil water content, pH, and soil organic matter, which are key factors driving plant species diversity in the latosol gully erosion area. This indicates that *A. mangium* pure and mixed forests generally have favorable soil physicochemical properties and can play an important role in improving the ecological environment during vegetation restoration. These findings contribute novel empirical evidence to support the selection of *A. mangium* pure and mixed forests for future ecological restoration projects to mitigate soil erosion and improve understory plant diversity and soil fertility in tropical gully erosion areas. They further provide a scientific basis for adaptive forest management aimed at enhancing both biodiversity conservation and soil fertility recovery.

## Figures and Tables

**Table 1:** The basic characteristics of the sampling plots.

Vegetation Restoration Forest Type	Dominant Tree Species	Average Tree Height (m)	Average Diameter at Breast Height (cm)	Tree Density (Trees·ha^−1^)	Canopy Density	Slope Gradient (°)
*E. robusta* forest	*E. robusta*	7.91 ± 2.14	8.64 ± 1.33	975	0.28	8~17
*A. mangium* forest	*A. mangium*	5.82 ± 1.72	13.28 ± 2.36	1033	0.78	16~21
*A. mangium–E. robusta* mixed forest	*A. mangium*, *E. robusta*	8.78 ± 2.15	8.70 ± 2.53	983	0.19	9~13
*A. mangium–E. robusta–S. pseudolima* mixed forest	*A. mangium*, *E. robusta*, *S. pseudolima*	8.76 ± 2.26	9.85 ± 2.24	367	0.27	8~12

Note: Data are presented as mean ± S.D.

**Table 2:** Species composition of understory plants in different vegetation restoration forest types.

Vegetation Restoration Forest Type	Vegetation Layer	Family	Genus	Species
*E. robusta* forest	Shrub layer	10	15	15
	Herbaceous layer	6	15	16
*A. mangium* forest	Shrub layer	6	7	7
	Herbaceous layer	4	10	10
*A. mangium–E. robusta* mixed forest	Shrub layer	7	10	10
	Herbaceous layer	7	16	17
*A. mangium–E. robusta–S. pseudolima* mixed forest	Shrub layer	5	6	6
	Herbaceous layer	6	17	18

**Table 3:** Soil physical properties of four vegetation restoration forest types.

Soil Physicochemical Properties	Vegetation Restoration Forest Type	Soil Layer				
		0–10 cm	10–20 cm	20–40 cm	40–60 cm	The Average Value
Soil bulk density (g·cm^−3^)	*E. robusta* forest	1.55 ± 0.06 a	1.61 ± 0.05 a	1.63 ± 0.10 a	1.66 ± 0.11 a	1.61 ± 0.05 ab
	*A. mangium* forest	1.44 ± 0.05 b	1.56 ± 0.06 a	1.60 ± 0.04 a	1.68 ± 0.05 a	1.57 ± 0.10 b
	*A. mangium–E. robusta* mixed forest	1.55 ± 0.06 a	1.62 ± 0.05 a	1.67 ± 0.04 a	1.66 ± 0.03 a	1.63 ± 0.05 a
	*A. mangium–E. robusta–S. pseudolima* mixed forest	1.53 ± 0.03 a	1.62 ± 0.04 a	1.66 ± 0.03 a	1.71 ± 0.03 a	1.63 ± 0.08 a
Soil capillary porosity (%)	*E. robusta* forest	34.96 ± 3.32 a	33.44 ± 4.18 a	30.72 ± 6.31 b	30.11 ± 5.17 ab	32.31 ± 2.29 b
	*A. mangium* forest	34.99 ± 2.34 a	36.58 ± 4.98 a	37.66 ± 1.89 a	34.42 ± 1.46 a	35.91 ± 1.48 a
	*A. mangium–E. robusta* mixed forest	34.80 ± 3.04 a	32.46 ± 1.81 a	31.28 ± 3.33 b	27.66 ± 1.22 b	31.55 ± 2.98 b
	*A. mangium–E. robusta–S. pseudolima* mixed forest	31.75 ± 3.29 a	34.85 ± 1.96 a	33.06 ± 2.28 ab	29.59 ± 3.07 b	32.31 ± 2.22 b
Total soil porosity (%)	*E. robusta* forest	37.97 ± 2.03 a	35.04 ± 4.23 b	32.24 ± 6.32 b	31.29 ± 4.96 b	34.14 ± 3.01 b
	*A. mangium* forest	37.77 ± 2.03 a	40.55 ± 2.97 a	40.01 ± 2.10 a	36.75 ± 1.88 a	38.77 ± 1.81 a
	*A. mangium–E. robusta* mixed forest	38.82 ± 3.36 a	35.27 ± 1.96 b	32.83 ± 4.13 b	29.45 ± 2.28 b	34.09 ± 3.95 b
	*A. mangium–E. robusta–S. pseudolima* mixed forest	38.54 ± 3.56 a	37.33 ± 1.98 ab	35.11 ± 2.86 ab	30.52 ± 2.76 b	35.38 ± 3.53 b
Soil water content (%)	*E. robusta* forest	3.41 ± 1.76 b	4.06 ± 1.49 c	4.61 ± 1.22 c	5.58 ± 0.93 b	4.42 ± 0.92 c
	*A. mangium* forest	3.22 ± 0.94 b	3.02 ± 0.46 c	3.95 ± 0.87 c	4.15 ± 0.47 c	3.59 ± 0.55 d
	*A. mangium–E. robusta* mixed forest	7.72 ± 1.19 a	6.45 ± 1.00 b	6.85 ± 0.59 b	6.72 ± 0.40 a	6.94 ± 0.55 b
	*A. mangium–E. robusta–S. pseudolima* mixed forest	7.51 ± 1.89 a	9.32 ± 1.66 a	8.55 ± 1.69 a	7.23 ± 1.87 a	8.15 ± 0.96 a
Soil maximum water-holding capacity (t·ha^−1^)	*E. robusta* forest	192.82 ± 7.15 a	180.94 ± 18.46 ab	164.94 ± 34.53 a	160.19 ± 27.49 ab	174.72 ± 14.98 b
	*A. mangium* forest	188.88 ± 10.13 a	195.27 ± 9.31 a	195.63 ± 8.87 a	181.69 ± 7.67 a	190.37 ± 6.56 a
	*A. mangium–E. robusta* mixed forest	197.67 ± 12.98 a	176.34 ± 9.80 b	170.51 ± 18.35 a	147.26 ± 11.40 b	172.95 ± 20.72 b
	*A. mangium–E. robusta–S. pseudolima* mixed forest	187.06 ± 8.34 a	183.99 ± 4.43 ab	180.60 ± 3.16 a	157.56 ± 10.07 b	177.30 ± 13.42 b
Soil effective water-holding capacity (t·ha^−1^)	*E. robusta* forest	170.65 ± 17.01 ab	166.14 ± 18.59 ab	151.17 ± 32.29 b	153.65 ± 27.75 ab	160.40 ± 9.47 b
	*A. mangium* forest	174.96 ± 11.67 a	179.63 ± 12.19 a	182.67 ± 10.14 a	167.98 ± 4.72 a	176.31 ± 6.39 a
	*A. mangium–E. robusta* mixed forest	172.13 ± 12.21 ab	157.60 ± 7.11 b	149.78 ± 11.24 b	136.59 ± 3.55 b	154.03 ± 14.86 b
	*A. mangium–E. robusta–S. pseudolima* mixed forest	155.00 ± 7.93 b	170.40 ± 6.17 ab	165.28 ± 11.42 ab	140.97 ± 16.50 b	157.91 ± 12.98 b

Data are mean ± S.D. Different lowercase letters indicate significant differences among vegetation restoration forest types at the same soil depth (*p* < 0.05).

**Table 4:** Soil chemical properties of four vegetation restoration forest types.

Soil Physicochemical Properties	Vegetation Restoration Forest Type	Soil Layer				
		0–10 cm	10–20 cm	20–40 cm	40–60 cm	The Average Value
pH	*E. robusta* forest	5.49 ± 0.07 a	5.62 ± 0.10 a	5.56 ± 0.17 a	5.63 ± 0.09 a	5.58 ± 0.06 a
	*A. mangium* forest	4.91 ± 0.22 c	4.95 ± 0.17 c	5.19 ± 0.12 b	5.18 ± 0.15 b	5.06 ± 0.15 c
	*A. mangium–E. robusta* mixed forest	5.50 ± 0.16 a	5.55 ± 0.17 a	5.61 ± 0.16 a	5.68 ± 0.16 a	5.59 ± 0.08 a
	*A. mangium–E. robusta–S. pseudolima* mixed forest	5.22 ± 0.09 b	5.13 ± 0.05 b	5.20 ± 0.06 b	5.14 ± 0.09 b	5.17 ± 0.04 b
Soil organic matter (g·kg^−1^)	*E. robusta* forest	10.64 ± 3.29 b	5.26 ± 2.95 c	2.57 ± 1.14 c	3.53 ± 1.58 c	5.50 ± 3.60 c
	*A. mangium* forest	20.73 ± 8.30 a	14.02 ± 3.59 a	9.61 ± 3.26 a	10.46 ± 3.73 a	13.71 ± 5.06 a
	*A. mangium–E. robusta* mixed forest	13.84 ± 2.82 b	10.03 ± 2.88 b	7.88 ± 1.62 ab	7.92 ± 1.92 ab	9.92 ± 2.80 b
	*A. mangium–E. robusta–S. pseudolima* mixed forest	8.49 ± 1.93 b	7.76 ± 1.88 bc	5.92 ± 2.45 b	5.85 ± 1.33 bc	7.01 ± 1.33 c
NH_4_^+^-N (mg·kg^−1^)	*E. robusta* forest	38.35 ± 3.25 c	37.36 ± 1.25 c	33.40 ± 0.67 c	34.32 ± 2.20 c	35.86 ± 2.37 d
	*A. mangium* forest	47.49 ± 9.96 b	40.15 ± 6.70 c	41.06 ± 7.56 c	46.50 ± 11.03 b	43.80 ± 3.73 c
	*A. mangium–E. robusta* mixed forest	70.01 ± 5.82 a	67.88 ± 5.63 a	61.76 ± 4.92 a	59.25 ± 1.34 a	64.73 ± 5.05 a
	*A. mangium–E. robusta–S. pseudolima* mixed forest	53.21 ± 5.76 b	54.62 ± 9.92 b	53.91 ± 12.24 b	53.06 ± 10.02 ab	53.70 ± 0.72 b
NO_3_^−^-N (mg·kg^−1^)	*E. robusta* forest	1.21 ± 0.59 b	0.74 ± 0.30 b	0.64 ± 0.36 b	0.74 ± 0.36 b	0.83 ± 0.26 b
	*A. mangium* forest	2.02 ± 0.66 b	1.62 ± 0.85 a	0.54 ± 0.21 b	0.72 ± 0.32 b	1.23 ± 0.71 b
	*A. mangium–E. robusta* mixed forest	4.79 ± 3.73 a	1.47 ± 1.05 ab	1.09 ± 0.49 b	3.77 ± 2.77 a	2.78 ± 1.79 a
	*A. mangium–E. robusta–S. pseudolima* mixed forest	3.06 ± 0.43 ab	1.70 ± 0.56 a	2.74 ± 1.68 a	1.94 ± 1.69 b	2.36 ± 0.64 a

Data are mean ± S.D. Different lowercase letters indicate significant differences among vegetation restoration forest types at the same soil depth (*p* < 0.05).

## Data Availability

The data that support the findings of this study are available from the corresponding author upon reasonable request. The data are not publicly available due to institutional policies.

## References

[1] Xia J., Zhang L., Ge P., Lu X., Wei Y., Cai C., Wang J. (2022). Structure degradation induced by wetting and drying cycles for the hilly granitic soils in collapsing gully erosion areas. Forests.

[2] Tu Z., Chen S., Chen Z., Ruan D., Zhang W., Han Y., Han L., Wang K., Huang Y., Chen J. (2023). Hydrological properties of soil and litter layers of four forest types restored in the gully erosion area of latosol in South China. Forests.

[3] Zheng J., Wan F., Cai Y., Liu J., Wang D., Guo X., Chen B. (2026). Multi-model machine learning mapping of gully erosion susceptibility in the Heihe region of the Xiaoxingán Mountains, China. Remote Sens..

[4] Deng L., Shangguan Z., Li R. (2012). Effects of the grain-for-green program on soil erosion in China. Int. J. Sediment Res..

[5] Zhao W.Q., Liu Y., Luo M.L., Wang Y.F., Lv Y.H. (2016). Effect of revegetation on soil erosion in small watershed of the Loess Plateau. J. Soil Water Conserv..

[6] Xu X.L., Ma K.M., Fu B.J., Liu X.C., Huang Y., Qi J. (2006). Research review of the relationship between vegetation and soil loss. Acta Ecol. Sin..

[7] Fang Y., Wei Q., Zhao J., Wan B.H., Yan X.J., Xiao J.J., Chen L.H., Han M.Q., Zhu P. (2022). Plant diversity in excavated slopes of different vegetation restoration stages in arid valleys of the Chengdu-Lanzhou Railway. Chin. J. Appl. Environ. Biol..

[8] Wang L. (2020). Effects of different stand densities on community structure and species diversity of *Pinus tabulaeformis* plantation. Ecol. Environ. Sci..

[9] Wen B.J., Duan G.H., Wen Z.M. (2023). Coupling effects of biodiversity with topographic and soil factors on slope runoff-sediment yield. Acta Agrestia. Sin..

[10] Bastin J.-F., Finegold Y., Garcia C., Mollicone D., Rezende M., Routh D., Zohner C.M., Crowther T.W. (2019). The global tree restoration potential. Science.

[11] Wang R.H., Ge X.M., Tang L.Z. (2014). A Review of Diversity, Biomass and Nutrient Effect of Understory Vegetation. World For. Res..

[12] Ammer C. (2019). Diversity and forest productivity in a changing climate. New Phytol..

[13] Zhu H., Tan Y.H. (2022). Flora and vegetation of Yunnan, Southwestern China: Diversity, origin and evolution. Diversity.

[14] Han L., Liu Y., Liu J., Kang H.L., Liu Z., Tuo F., Gan S.A., Ren Y.X., Yi C.H., Hu G.M. (2025). Microtopography affects the diversity and stability of vegetation communities by regulating soil moisture. Water.

[15] Li M.J., He Z.S., Jiang L., Gu X.G., Jin M.R., Chen B., Liu J.F. (2021). Distribution pattern and driving factors of species diversity and phylogenetic diversity along altitudinal gradient on the south slope of Daiyun Mountain. Acta Ecol. Sin..

[16] Fujii S., Mori A.S., Koide D., Makoto K., Matsuoka S., Osono T., Isbell F. (2017). Disentangling relationships between plant diversity and decomposition processes under forest restoration. J. Appl. Ecol..

[17] Wu A.C., Deng X.W., Ren X.L., Xiang W.H., Zhang L., Ge R., Niu Z.G., He H.L., He L.J. (2018). Biogeographic patterns and influencing factors of the species diversity of tree layer community in typical forest ecosystems in China. Acta Ecol. Sin..

[18] Yang X.M., Feng Q., Zhu M., Zhang J.T., Yang L.S., Li R.L. (2024). The impact of artificial restoration of alpine grasslands in the Qilian Mountains on vegetation, soil bacteria, and soil fungal community diversity. Microorganisms.

[19] Pohl M., Alig D., Körner C., Rixen C. (2009). Higher plant diversity enhances soil stability in disturbed alpine ecosystems. Plant Soil.

[20] Ma X.Y., Tian X., Guo Y.T., Lin Y.M. (2023). Effect of vegetation restoration on soil stoichiometry characteristics in southwestern China based on meta-analysis. Chin. J. Appl. Environ. Biol..

[21] Wingfield M.J., Brockerhoff E.G., Wingfield B.D., Slippers B. (2015). Planted forest health: The need for a global strategy. Science.

[22] Wang M.Z., Bi H.J., Jin S., Liu J., Liu Y.H., Wang Y., Qi J.Q., Hao J.F. (2019). Effects of stand density on understory species diversity and soil physicochemical properties of a *Cupressus funebris* plantation in Yunding Mountain. Acta Ecol. Sin..

[23] Chen H.L., Gou M.M., Liu C.F., Lei L., Hu J.W., Zhu S.F., Hu R.Y., Xiao W.F. (2024). Relationship between understory plant diversity and soil physicochemical properties of different aged *Pinus massoniana* plantations in hilly region of central Hubei province. Ecol. Environ. Sci..

[24] Sun W.Y., Yu X.X., Jia G.D., Wang X., Meng J., Niu Y.M. (2024). Correlation between soil factors and understory plant diversity in different Plantations in the semi-arid regions of Inner Mongolia. J. Northeast For. Univ..

[25] Ding L., Lin Q., Gao X.Y. (2025). Effects of different regeneration tree species on the growth of *Larix kaempferi* plantation forests, the diversity of understory plants and the soil properties. J. Northeast For. Univ..

[26] Meng X.Y., Fan S.X., Dong L., Kong X.Y., Wang M.M., Li K., Wang W.L. (2023). Correlation between understory plant diversity and soil factors in Beijing urban forests. J. Northeast For. Univ..

[27] Huang X., Yu X.X., Jia G.D., Chang X.M., Sun L.B. (2025). Effects of stand density on understory vegetation and soil properties in *Populus simonii* plantations. Chin. J. Appl. Ecol..

[28] Lv K.T., Zhou M.L., Ding Y., Zang R.G., Yao J., Luo Y.S., Yan D.F. (2023). Regeneration characteristics and influencing factors of woody plant on natural evergreen secondary broad-leaved forests in the subtropical, China. Glob. Ecol. Conserv..

[29] Hu Y.W., Shi Z.G., Liu C., Xu Q.T., Zhang J.J. (2023). Effects of stand densities on understory vegetation diversity and soil physicochemical properties of *Robinia pseudoacacia* forest in loess region of western Shanxi Province. Chin. J. Ecol..

[30] Zhen Q., Wang B.T., Zhao Y., Wang X.H., Ma B.M., Gao H.P. (2020). Evaluation of plantation quality based on soil physico-chemical properties and plant diversity in the loess area of western Shanxi province. Sci. Soil Water Conserv..

[31] Li J.J., Yang L., Fan M.C., Shangguan Z.P. (2022). Plantation vegetation restoration enhances the relationship between rhizosphere microbial diversity and soil multifunctionality. Land Degrad. Dev..

[32] Tang B.A., Yu Z.Y., Chen C.F., Yuan J.P., Fan Y.Q., Qiu P.H., Li C.E. (2010). Analysis on ecogeographical characteristics of soil animal groups of *Eucalyptus robusta* artificial woodland in Mahuangling watershed in Hainan Island. Geogr. Res..

[33] Huang Y.P., Hong D.W., Han Y.J., Wang K., Yu Y., Chen S.Y., Tu Z.H. (2025). The recovery process of litter water-holding capacity in different mixed forests in typical soil and water loss areas of Hainan Province. Soil Water Conserv. China.

[34] Chen S., Huang Y., Yan M., Han Y., Wang K., Chen Z., Ruan D., Yu Y., Tu Z. (2024). Differential Water Conservation Capacity in Broadleaved and Mixed Forest Restoration in Latosol Soil-Eroded Region, Hainan Province, China. Plants.

[35] (2006). Soil Testing Part 2: Method for Determination of Soil pH.

[36] (2006). Soil Testing Part 6: Method for Determination of Soil Organic Matter.

[37] (2023). Soil Quality—Determination of Nitrate, Nitrite and Ammonium in Soils—Extraction with Potassium Chloride Solution and Determination with Manual Method.

[38] Deng C., Huang Z., Zhang X., Zhao H., Jiang S., Ren Y. (2022). Correlation between Vegetation Structure and Species Diversity in Traditional Villages in Karst Topographic Regions of the Zunyi City, China. Plants.

[39] Gou J.H., Wu S.X., He C., Hu C.H., Yang D., Liu S.J. (2021). The relationship between soil water contents in different sections of gully and plant diversity in Yuanmou Dry-hot Valley. Southwest China J. Agric. Sci..

[40] Mo Y.F., Wang J.Y., Chen L., Wei G.Y., Yang M. (2022). Effects of different mixed models on growth and plant diversity in *Eucalyptus* plantations. Southwest China J. Agric. Sci..

[41] Zhang G.Z., Gu J.L., Wang F., Chen Z.T., Gan J.J., Xu M.S. (2026). Floristic composition, species diversity, and influencing factors of vegetation in typical beach ecosystems of Qinhuangdao. Chin. J. Appl. Environ. Biol..

[42] Du S., Yu X.B., Shi M.Y., Zhou H., Chen H.H., Huang H., Wu J.Q. (2023). Characteristics of understory plant diversity of *Eucalyptus* plantation in Hainan. China J. Trop. Crops.

[43] Sui X.R., Zhao Q.J., Zhou X.Q., Chen J., Chen J., Peng Q., Zhang Z.X. (2024). Response of plant diversity under *Platycladus orientalis* plantation to canopy density in Xuzhou. J. Nanjing For. Univ. Nat. Sci. Ed..

[44] Ao L., Zhao M.C., Li X., Sun G.Y. (2022). Different urban forest tree species affect the assembly of the soil bacterial and fungal community. Microb. Ecol..

[45] Wang G.H., Wang Y.J., Huang L.S., Jin C.S., Liu L.Y. (2014). Study on plant diversity in erosion gullies in *Larix principis-rupprechtii* plantations. Hebei For. Fruit. Res..

[46] Jiang L., Zhang P.F., Wang J., Qin X.P., Wang H.L., Zhou S.X., Huang C.D. (2024). Effects of constructing mixed forests on understory shrub and herb species diversity after clear-cutting of Eucalyptus grandis pure forest. Chin. J. Appl. Environ. Biol..

[47] Zeng R.J., Yang X.B., Li D.H., Wang Q., Wang H., Xia D. (2024). Relationship between understory plant diversity and environmental factors in Eucalyptus robusta and *Acacia mangium* forests. Nat. Sci. J. Hainan Univ..

[48] He Z.W., Zha X., Huang S.Y. (2015). The causes and ecological restoration measures of erosion and degradation in the red soil hilly erea of Southern China. Subtrop Soil Water Conserv..

[49] Wu Q., Jiang Y.R., Li Q.Y., Xing X.Y., Li S., Yang L., Bai T. (2025). Plant diversity in urban shaded habitats with different canopy densities. J. Chin. Urban For..

[50] Liu J.J., Lv Q., Shen Y., Liu J.L., Hou Q., Chen Y.Q., Fan C., Li X.W. (2021). Early effects of target tree management on plant diversity and soil physicochemical properties in a *Cunninghamia lanceolata* plantation. Chin. J. Appl. Environ. Biol..

[51] Liu X.E., Su S.P., Li Y., Wang W. (2021). Study on the ecological benefits of a plantation mixed forest model in the Loess Plateau. Arid Zone Res..

[52] Yuan Z.X., Guan Q.W., Li J.J., Han M.H., Jin X.M., Chen X. (2022). Effect of Various Vegetation Restoration Types on Soil Physio-chemical Properties. J. Northeast For. Univ..

[53] He D.Z. (2017). The Effects of different residue retention densities on the growth of *Acacia mangium* and soil physical and chemical properties. J. Green Sci. Technol..

[54] Yuan L.M., Yang Z.G., Xue B., Gao H.Y., Han Z.R.G.T. (2022). Heterogeneity of soil moisture of blowouts in HulunBuir grassland. Arid Zone Res..

[55] Wang Y.J., Chen Y.M., Sun Y.R., Zhao M. (2023). Study on physical and chemical properties and erodibility of soil in different parts of erosion gully in the mountainous area of South Ningxia. J. Soil Water Conserv..

[56] Wang Y.J., Chen Y.M., Sun Y.R., Zhao M. (2023). Plant diversity and soil nutrients in different parts of erosion gully in loess hilly region of Southern Ningxia. Res. Soil Water Conserv..

[57] Yan S., Xia F., Wei W., Wang J.L., Wu H.Y., Ran L.L., Xue Y.Y., Shi H., Zheng S.K., Wang J.Q. (2025). Differences along an erosion gradient in alpine meadow plant community diversity and factors influencing diversity. Acta Pratacult Sin..

[58] Yan W.M., Sun B., Pei N.C., Wang X., Li F.F., Luo X.H., Zou B. (2019). Correlation analyses on plant diversity and soil physical-chemical properties between evergreen broad-leaved plantations and natural secondary forests in North Guangdong, China. Ecol. Environ. Sci..

[59] Chen Q.H., Wen Y.G., Zhou X.G., Wang L., Sun D.J., Huang X.G., Tang L., Xu J.M., Huang Y.J., Deng S.H. (2023). Effects of different regeneration methods on understory plant diversity after *Eucalyptus* plantations replaced *Pinus massoniana* plantations. Guangxi Sci..

